# Raman Spectroscopy—A Novel Method for Identification and Characterization of Microbes on a Single-Cell Level in Clinical Settings

**DOI:** 10.3389/fcimb.2022.866463

**Published:** 2022-04-22

**Authors:** Katarina Rebrosova, Ota Samek, Martin Kizovsky, Silvie Bernatova, Veronika Hola, Filip Ruzicka

**Affiliations:** ^1^ Department of Microbiology, Faculty of Medicine of Masaryk University and St. Anne’s University Hospital, Brno, Czechia; ^2^ Institute of Scientific Instruments of the Czech Academy of Sciences, Brno, Czechia

**Keywords:** Raman spectroscopy, Raman tweezers, identification of microorganisms, antimicrobial resistance, microfluidic devices, magnetic beads, diagnostics

## Abstract

Rapid and accurate identification of pathogens causing infections is one of the biggest challenges in medicine. Timely identification of causative agents and their antimicrobial resistance profile can significantly improve the management of infection, lower costs for healthcare, mitigate ever-growing antimicrobial resistance and in many cases, save lives. Raman spectroscopy was shown to be a useful—quick, non-invasive, and non-destructive —tool for identifying microbes from solid and liquid media. Modifications of Raman spectroscopy and/or pretreatment of samples allow single-cell analyses and identification of microbes from various samples. It was shown that those non-culture-based approaches could also detect antimicrobial resistance. Moreover, recent studies suggest that a combination of Raman spectroscopy with optical tweezers has the potential to identify microbes directly from human body fluids. This review aims to summarize recent advances in non-culture-based approaches of identification of microbes and their virulence factors, including antimicrobial resistance, using methods based on Raman spectroscopy in the context of possible use in the future point-of-care diagnostic process.

## Introduction

Microorganisms play irreplaceable roles in human existence—we can find them everywhere. In fact, human bodies include more microbial cells than their own cells (Sender et al., 2016). At the same time, it is estimated that approximately 1400 pathogens (including bacteria, fungi, viruses, protozoa, and helminths) can cause infections in humans (Balloux and van Dorp, 2017; Franco-Duarte et al., 2019).

Despite enormous progress in medicine during the last decades, accurate and rapid species-level identification of pathogens causing infections and their virulence factors (including antimicrobial resistance and ability to form biofilms) still poses a challenge. It is important to accent that timely identification and characterization of pathogens is essential for choosing a suitable tailored antimicrobial treatment and proper management of patients. This, in turn, leads to the shortening of hospital stays, reducing costs and time to adequate treatment, increasing the wellbeing of patients, reducing the spread of antimicrobial resistance, and, above all, saving the lives of many patients.

## Identification of Microorganisms

Basically, we can divide existing identification methods into two groups: culture-based and direct approaches (without cultivation). Major advantages and disadvantages of established methods are summarized in the **Table 1**.

**Table 1:** Summary of current advantages and disadvantages of Raman spectroscopy in microbiology.

Raman spectroscopy and microbial analyses	
Advantages	Disadvantages
rapid sensitive non-destructive non-invasive	no commercial database for microbial identification
microbes remain viable after the analysis and can be used for further testing	need for standardization (data presented in manuscripts are group-specific and custom-tailored)
highly reproducible within a device simple sample preparation	
no costly consumables necessary	relatively expensive device
allowing detection of virulence factors	need for trained personnel and possible automatization
allowing single-cell level analyses: applicable for non-culturable microbes, no need for cultivation	cultivation is necessary or can be replaced by separation methods

Culture-based approaches are widely used in clinical diagnostics; they could be considered “golden standards”. Cultivation provides large amounts of microbial cells for further testing and offers a way to separate different microbes from a mixed culture. This allows the application of various identification and characterization methods separately or in combinations. However, it also makes the culture-based methods relatively time-consuming, expensive, and labor-demanding. Methods commonly used in clinical diagnostics include biochemical testing and mass spectroscopy-based methods. Due to the ever-growing problem of antimicrobial resistance, additional testing of antimicrobial susceptibility (AST) is often required. Conventional AST methods include disk diffusion, gradient diffusion, microdilution, and E-test—all of them require the cultivation step (Khan et al., 2019).

### Mass Spectrometry

As an alternative to biochemical testing, matrix-assisted laser desorption/ionization-time-of-flight mass spectrometry (MALDI-TOF MS) has become a revolutionary, widely used tool in numerous clinical diagnostic laboratories. The method is based on the ionization of chemical compounds and measurements of their mass to charge (m/z) ratio. These create a specific microbial fingerprint (a peptide mass fingerprint) allowing identification upon comparison with databases. The whole process takes only minutes and can distinguish even between the most closely related microbial species (Bader et al., 2011; Hendrickx et al., 2011; Lee et al., 2017; Rychert, 2019) and detect antimicrobial resistance (Burckhardt and Zimmermann, 2018; Florio et al., 2020). However, the procedure involves multiple-step-sample preparation and relatively costly consumables. The expensive MALDI-TOF MS device makes the method unaffordable for laboratories in developing countries (Yonetani et al., 2016; Peng et al., 2019).

### Direct Methods

Direct methods, besides characterization of microbes and screening, can be used for the identification of microbes in mixed samples as well as for identification of non-culturable microbes (Ramamurthy et al., 2014; Franco-Duarte et al., 2019). They are predominantly based on microscopy, serology, or molecular analyses and do not require cultivation.

### Microscopy

Microscopy techniques (bright-field microscopy, dark-field microscopy) can give some indication of the presence of microbes in a sample (Franco-Duarte et al., 2019). A combination of microscopy techniques with other tools/procedures can be used to increase the identification power. Examples include fluorescent dyes (Amann and Fuchs, 2008; Sabnis, 2015), scanning electron microscopy (SEM) (Relucenti et al., 2021), transmission electron microscopy (TEM) (McCutcheon and Southam, 2018), confocal microscopy (CLSM) (Cardinale et al., 2017), and atomic force microscopy (ATM) (James et al., 2016). These methods are also considered valuable in biofilm studies. A disadvantage of direct microscopy methods is an unspecific result not allowing accurate identification unless combined with additional procedure/tools, which makes the process costly and time-demanding.

### Serological Methods

Serological methods used in clinical diagnostics include the detection of antigens and antibodies. They are usually highly specific and must be ordered in a goal-directed manner (e.g., confirmation or exclusion of certain infectious agents). The positivity of antibody tests may be delayed due to the dynamics of antibody production in the human body. Besides, the immune response of the hosts usually has polyclonal nature and is influenced by genetic factors as well as environmental factors. Therefore, the reaction of a patient´s serum with an analytical system is not precisely predictable and there might be some variations. During the detection of antigens, antigenic variations (leading to different serotypes) might cause problems (Fierz, 2003).

### Molecular Methods

The advent of the “genomic era” brought an astounding array of techniques incredibly useful for the characterization of biological materials and organisms, including microbes (Spratt, 2004). The development of polymerase chain reaction (PCR) in 1983 was a real breakthrough in (clinical) microbiology. Later on, real-time PCR (RT-PCR) was developed and brought a new wind into diagnostics improving the speed, sensitivity, and specificity of microbial detection (Mackay, 2004). Also, 16S rRNA, 16S – 23S rRNA (bacteria), and 18S rRNA (eukaryotes) PCR-sequencing can be a useful tool for identifying microorganisms combining PCR amplification of 16S (18S) rRNA gene, which is highly specific to each microbial species, and its subsequent sequencing (Reller et al., 2007; Singhal et al., 2015; Peker et al., 2019). When 16S rRNA gene is identical, species identification could rely on other conserved genes, such as gyrA, gyrB, rpoB, tuf, and heat shock proteins (Franco-Duarte et al., 2019). Although PCR-based methods can detect pathogens at early stages of infection and do not require cultivation, clinical samples often contain low numbers of microbial cells, complicating their capturing. They require preprocessing before the PCR reaction (incl. removal of PCR inhibitors, extraction of maximum microbes from the sample without contamination, isolation of nucleic acids). Furthermore, those methods detect the only presence of nucleic acid (or its part), which can be misleading since the human body contains large amounts of microbial genetic material (Franco-Duarte et al., 2019; Kubina and Dziedzic, 2020).

Modern technologies allow miniaturization and automatization of the methods and building more efficient approaches, such as next-generation sequencing (NGS). NGS allows parallel sequencing of enormous numbers of whole genomes or parts of nucleic acids at once, providing reliable identification of microorganisms at the nucleic acid level (Sabat et al., 2017; Boers et al., 2019). To overcome the low numbers of pathogens present in a sample, PCR amplification can be used. Compared with other widely used methods, disadvantages of using NGS in clinical diagnostics of infections include high costs and lower sensitivity, or more specifically, higher recovery of clinically unimportant microorganisms, and often too general identification (e.g., phylum level) (Lee et al., 2020).

### New Approaches

We can conclude that all the methods mentioned above have advantages and disadvantages. Thus, different methods might be suitable or combined for different types of infections/expected causative agents. To shorten the time necessary for identification, many approaches have been proposed. Their main goals include shortening/skipping cultivation, shortening the time and reducing consumables needed for the identification, automatizing the process, and/or adding further information about a sample (e.g., virulence factors). These approaches include but are not limited to certain protocols for MALDI-TOF MS (Drancourt, 2010; Meex et al., 2012; Hoyos-Mallecot et al., 2014; Idelevich et al., 2014; Machen et al., 2014; Verroken et al., 2015; Zhou et al., 2017), infrared spectroscopy (FTIR) (Ojeda and Dittrich, 2012; Zarnowiec et al., 2015; Vogt et al., 2019), nuclear magnetic resonance (NMR) spectroscopy (Romaniuk and Cegelski, 2015; Palama et al., 2016), capillary electrophoresis (incl. capillary isoelectric focusing (Ruzicka et al., 2016; Xu and Sun, 2021), electrical field-flow fractionation (Saenton et al., 2000; Reschiglian et al., 2002), microfluidic devices (Kim et al., 2012; Zhou et al., 2019; Pérez-Rodríguez et al., 2022) and Raman spectroscopy (Franco-Duarte et al., 2019). Recently, Raman spectroscopy has been undergoing a boom in microbiology, as many studies suggest its potential for the identification of microbes, their virulence factors, detection of metabolic changes, and last but not least, single-cell analyses of microbial cells.

## Raman Spectroscopy

**Figure 1:**
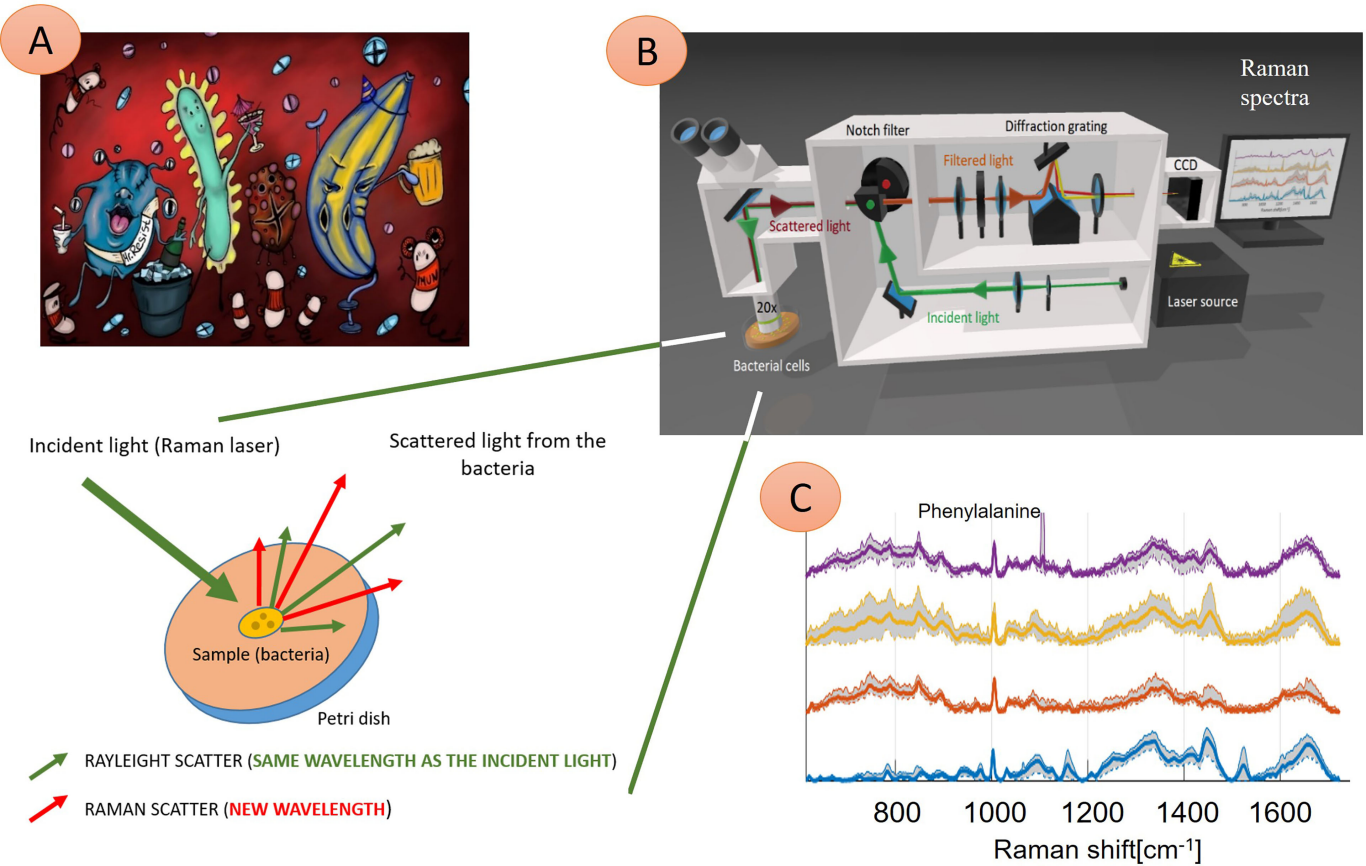
Illustration of four different microbial species interacting with the immune system and their Raman spectra. **(A)** Microbes are present in the human body, causing an infection. These pathogens can be identified using Raman spectroscopy technique **(B)**—here: probing laser (green) is focused on the sample (bacteria), and a small amount of light, which transports the chemical structure of analyzed bacteria, is reflected (red) and in the next step further analyzed. Consequently, four Raman spectra **(C)** show information about molecular bond vibrations of given bacteria, such as phenylalanine at 1005 cm^-1^. In this example, the naked eye can see differences between the spectra of four samples. Thus, these pathogens can be identified quickly (in minutes) to treat infection with tailored antibiotics. **(C)** Examples of Raman spectra: *Staphylococcus pasteuri* (violet curve), *Staphylococcus warneri* (yellow curve), *Streptococcus oralis* (red curve), *Staphylococcus sciuri* (blue trace).

Raman spectroscopy is an optical method based on inelastic scattering of monochromatic light. In general, when a light beam (laser beam) reaches an object (particles), most of the light is scattered elastically (energy after deexcitation is equal to Rayleigh scattering), part of the beam passes through, part of the beam is absorbed. The last tiny part (approximately 10^-5^%) is inelastically scattered (**Figure 1**)—the energy of photons in the beam changes upon interaction with molecular vibrations in a sample and leads to a momentary distortion of electrons in a bond of a molecule. It means that the molecule has an induced dipole and is temporarily polarized. Upon returning to its normal state, the radiation is reemitted (Raman scattering). If a photon passes a part of its energy on a molecule, its frequency gets lower, and the vibrational energy of the molecule participating in a collision gets higher (Stokes Raman scattering). Thanks to thermal energy, it is also possible that a molecule is in an excited state. Then, a photon can gain energy from the molecule: the energy and the photon’s frequency gets higher, the energy of the molecule gets lower (anti-Stokes Raman scattering) (Das and Agrawal, 2011). Therefore, molecular vibrations in a sample play an essential role in Raman scattering: to be Raman active, a molecule must undergo a change in polarizability of an electron cloud around the molecule (a tendency of the electron cloud to be distorted from its original position) during a vibration. The polarizability of molecules decreases with increasing electron density (shorter and stronger bonds). The intensity of the Raman spectrum is dependent on the change of polarizability. Therefore, the most intensive Raman spectra can be acquired from symmetric valence vibrations (Smith and Dent, 2004). To conclude, we can say that the basic principle of Raman spectroscopy is tracking of scattered electrons´ energetic changes against the energy of photons from a source of monochromatic light “mirroring” chemical bonds present in the sample.

This can be useful in various scientific and industrial fields ranging from archeology arts (Ziemann and Madariaga, 2021) and food industry (Weng et al., 2019), pharmacy (Vankeirsbilck et al., 2002), life sciences (McCreery, 2000; Pimenta et al., 2007; Butler et al., 2016; Kuhar et al., 2018; Li et al., 2020; Wang et al., 2020; Pezzotti, 2021) to medicine. Examples of medical applications include measurements of inflammatory markers including C-reactive protein (Bergholt and Hassing, 2009; Neugebauer et al., 2014), measurements of blood and urine chemicals (Qi and Berger, 2007), measurements of blood coagulation (Poon et al., 2012), determination oxygen saturation in live tissues (Das and Agrawal, 2011), tissue engineering (Ember et al., 2017), *in vivo* and *in vitro* diagnostics of various cancers (Chan et al., 2008; Harvey et al., 2008; Dochow et al., 2013; Taleb et al., 2013; Kong et al., 2015; Auner et al., 2018), diagnostics of prenatal diseases (Kim et al., 2018), endometriosis (Parlatan et al., 2019), and osteomyelitis (Khalid et al., 2018). Raman spectroscopy also has a plethora of applications in clinical, experimental, environmental, and technical microbiology.

### Raman Spectroscopy in Microbiology

Raman spectroscopy appears to be a valuable tool for the identification of microorganisms (Maquelin et al., 2003; Samek et al., 2008; Almarashi et al., 2012; Kastanos et al., 2012; Schie and Huser, 2013; Neugebauer et al., 2015; Pahlow et al., 2015; Read and Whiteley, 2015; Tien et al., 2016; Rebrošová et al., 2017; de Siqueira e Oliveira et al., 2021; Rebrošová et al., 2022), even in mixed samples (Yogesha et al., 2019). The identification can be performed from colonies grown on solid agar plates, microcolonies (Choo-Smith et al., 2001; Mathey et al., 2015), or microorganisms in liquid media (Schuster et al., 2000; Samek et al., 2010; Avci et al., 2015; Kotanen et al., 2016; Nakar et al., 2022; Rebrošová et al., 2022) and microbial spectra are highly reproducible within a device (Mlynáriková et al., 2015). Furthermore, Raman spectroscopy can be used for the characterization of microbial virulence factors, including antimicrobial resistance (Wulf et al., 2012; Bernatová et al., 2013; Dekter et al., 2017; Rousseau et al., 2021; Nakar et al., 2022) and the ability to form a biofilm (Samek et al., 2008; Samek et al., 2014; Liu et al., 2014; Hrubanova et al., 2018; Keleştemur et al., 2018; Rebrošová et al., 2019). There are some Raman studies of phenotypic changes caused by exposure to environmental stimuli, including antibiotics (Athamneh et al., 2014), alcohol (Zu et al., 2016), or metabolic stressors (Tanniche et al., 2020). Raman spectroscopy was successfully used to quantify microbes in a sample, too (Escoriza et al., 2006). To gain a stronger signal, the Raman signal can be amplified using surface-enhanced Raman spectroscopy (SERS), which is widely used in microbiological studies (Samek et al., 2021). Recently, there was significant progress in single-cell analyzes employing Raman spectroscopy and other variations of Raman spectroscopy, allowing to skip the cultivation step. The most frequently used approaches are summarized below.

### Centrifugation

A commonly used method for separating microbes from liquid media/samples is centrifugation. Published works consider centrifugation+Raman spectroscopy to be promising for identification of microbes from human body fluids, namely ascitic fluid (Kloß et al., 2015b), sputum (Kloß et al., 2015a), artificial bronchoalveolar lavage (Wichmann et al., 2021), and urine (Schröder et al., 2015; Rebrošová et al., 2022). Premasiri et al. showed a possibility to combine centrifugation and SERS to identify pathogens and their antimicrobial susceptibility (Premasiri et al., 2017). Together with filtration lysis and SERS, centrifugation was used to identify pathogens from human serum (Kotanen et al., 2016).

Moreover, centrifugation+SERS showed a possibility of identifying *Chlamydia trachomatis* and *Neisseria gonorrhoeae* and characterizing their extra-cellular metabolomics (Chen et al., 2018).

### Magnetic Beads

Magnetic beads are widely used for separation and isolation during bioprocessing, especially for the isolation of nucleic acids. However, the mechanism itself allows magnetic separation to be applied on various samples: magnetic separation relies upon forces induced in magnetically susceptible materials by magnetic fields (Schwaminger et al., 2019). In biology, primarily magnetic beads coated with synthetical or biological polymers (including antibodies) capable of capturing the target molecules/cells are used. Target molecules/cells bind to a polymer. Afterward, the whole complex (magnetic carrier with polymer + target molecule/cell) is captured by applying magnetic force (Berensmeier, 2006).

Kusić et al. successfully used magnetic beads coated with *Legionella* spp. specific polyclonal immunoglobulins for isolating single *Legionella* sp. cells from biofilm and subsequently identifying them with Raman spectroscopy (Kusić et al., 2014). Kearns et al. developed a bionanosensor based on magnetic separation and SERS, which can be used to identify microbes in concentrations as low as 10^1^ CFU/mL in less than one hour (Kearns et al., 2017). Hu et al. showed a possibility of capturing *Candida* sp. cells from serum and characterizing them using SERS (Hu et al., 2021). Li et al. used polyethyleneimine-modified magnetic microspheres (Fe_3_O_4_@PEI) and SERS for bacterial identification and antimicrobial resistance determination from 77 blood samples (Li et al., 2019). A combination of SERS and immunomagnetic beads can also be used to detect *Clostridium botulinum* toxins A and B (Kim et al., 2019). Since magnetic separation is commonly used for nucleic acid isolation, Hwang et al. applied this method to isolate bacterial genomic DNA and identify it using SERS and fluorescent assay favoring SERS by means of sensitivity (Hwang et al., 2021). Detection of bacterial DNA by SERS using streptavidin-coated magnetic particles was also proved by Qun et al. with a limit of detection of 5 pM (Qun et al., 2015).

### Dielectrophoresis

Dielectrophoresis (DEP) is defined as a movement of dielectric particles through a medium in response to a non-uniform electric field. A particle becomes polarized, and due to the difference in electric field strength on the two sides of the particle, the particle is moved in the electric field gradient region by net dielectrophoretic force (Tay et al., 2009). This effect can be used for the separation of particles (Fernandez et al., 2017) and is widely used for enrichment and isolation of microbial cells before analysis at a single-cell level (Fernandez et al., 2017; Zhang et al., 2019; Sarno et al., 2021; Weber et al., 2021). Some groups recently suggested its possible combination with Raman spectroscopy for single-cell microbial analyses (Neugebauer et al., 2015). Chen et al. showed the efficiency of DEP-based microfluidic chip for Raman detection and measurements of *Shewanella oneidensis* cells in HEPES buffer (Chen et al., 2018). From clinically relevant applications, Cheng et al. used DEP with SERS to isolate and identify bacteria in isotonic solution with human blood cells, proposing rapid detection of microbes in human blood from 12h blood cultures (Cheng et al., 2014). To identify pathogens causing urinary tract infections faster, Schröder et al. proposed using a combination of DEP and Raman spectroscopy to identify *Escherichia coli* and *Enterococcus faecalis* in human urine, providing results in 35 minutes (Schröder et al., 2013).

### Optical Tweezers

Optical tweezers—a Nobel price-winning (2018) groundbreaking invention by Alfred Ashkin—use single-beam gradient force to hold particles/micro-objects (incl. microbial cells) in place and manipulate them (Ashkin, 1997; Wu et al., 2017). With an optical trap, living cells suspended in a liquid cultivation medium can be immobilized in a solution using the forces generated by a tightly focused laser beam. Combined with Raman spectroscopy (usually termed Raman tweezers), it has found numerous applications, especially in analytical and physical chemistry. Raman tweezers is currently starting to be applied in cell and molecular biology. It allows single-cell analyses of microbes, for example, direct identification of microbes from liquid samples, including wastewater (Cui et al., 2021) and human urine (Rebrošová et al., 2022), in less than 10 minutes. This combination can also be used to detect antibiotic resistance (Bernatová et. al 2014; Pilát et al., 2018; Bernatová et al., 2021) and an ability to form a biofilm (Samek et al., 2015). Moreover, it was used to describe metabolic changes in microbes (Singh et al., 2006; Huang et al., 2007) and bacterial lysis (Chen et al., 2009).

### Challenges

Current advantages and disadvantages of the Raman spectroscopy for microbiology are summarized in the **Table 1**.

Unambiguously, Raman spectroscopy offers a streamlined, fast, non-destructive, and non-invasive approach for identifying microbes and their virulence factors suitable for clinical diagnostics. Compared to conventional and molecular methods, sample preparation is easy and does not necessarily require expensive consumables. Moreover, multiple methods were proposed to isolate or enrich microbes, allowing their single-cell analyses and non-culture-based detection and identification.

However, getting Raman spectroscopy to clinical laboratories might have a long-time coming. At the moment, there are no commercial and/or well-annotated databases of microbial Raman fingerprints, as discussed by Wang et al. (Wang et al., 2021). To make comparisons of acquired Raman spectra across various instruments, one should consider that the quantum efficiency of the given detector and optical elements depends on a wavelength. Therefore, acquired data should be corrected according to an instrument response profile. Ideally, a spectral sensitivity curve should be used (Rebrošová et al., 2017). Thus, Raman spectra presented in different studies are group-specific and custom-tailored, which makes data standardization complicated (Lorenz et al., 2017). Nonetheless, no large-scale studies were performed to compare microbial Raman spectra from different groups.

Microbial studies often employ the SERS technique. Again, there is a problem with the standardization of SERS microbial detection protocols (Witkowska et al., 2020; Samek et al., 2021). Although SERS provides a stronger signal than conventional Raman spectroscopy, several factors influence the signal enhancement and, consequently, the final spectrum. These may include types of culture media, culturing conditions, sample preparation method, and interactions between SERS substrate and individual microbial cells (Witkowska et al., 2020; Samek et al., 2021).

Other problems are (currently) relatively high input costs, the need for complicated instrumentation, and specialized operators. Automatization and miniaturization would be beneficial for potential future clinical use.

If those problems are solved, Raman spectroscopy-based point-of-care device could start a revolution in clinical diagnostic microbiology in the future. Assuming its broad spectrum of applications in medicine, one device equipped with various diagnostic databases might become a complex diagnostic tool. From a microbiological point of view, Raman spectroscopy could be used for rapid pathogen identification and characterization of its virulence factors, which would, in turn, result in tailored antimicrobial treatment, reduced financial burdens associated with healthcare, and above all, improved patient management and reduced mortality from infections.

## Summary

Raman spectroscopy is an elegant optical method that could significantly contribute to rapid clinical diagnostics of infections. It allows the identification of microbes and detection of certain virulence factors, including antimicrobial resistance and biofilm formation. In combination with techniques for isolation/enrichment of microbes from a liquid sample, it could be used for rapid single-cell analyzes of microbial cells, even directly from human body fluids. Therefore, it could provide an effective solution for identifying microbes in the future.

## Author Contributions

KR, OS, MK participated in conceptualization and writing of the original draft. SB, VH, and FR participated in review and editing. All authors contributed to the article and approved the submitted version.

## Funding

This work was supported by the Grant Agency of Masaryk University (MUNI/A/1291/2021) and the Czech Health Research Council (NU21-05-00341).

## Conflict of Interest

The authors declare that the research was conducted in the absence of any commercial or financial relationships that could be construed as a potential conflict of interest.

## Publisher’s Note

All claims expressed in this article are solely those of the authors and do not necessarily represent those of their affiliated organizations, or those of the publisher, the editors and the reviewers. Any product that may be evaluated in this article, or claim that may be made by its manufacturer, is not guaranteed or endorsed by the publisher.
